# Metachronous hepatocellular carcinoma after partial response of advanced intrahepatic cholangiocarcinoma treated with radiotherapy combined with apatinib and camrelizumab: a case report

**DOI:** 10.3389/fonc.2026.1822063

**Published:** 2026-05-13

**Authors:** Yujuan Dai, Dachao Chen, Shufeng Wu, Yuehua Geng, XianYing Chen, Nana Zhang, Xiangbin Tan

**Affiliations:** 1 Department of Oncology, The 909th Hospital, School of Medicine, Xiamen University, Zhangzhou, Fujian, China; 2 Department of Pathology, The 909th Hospital, School of Medicine, Xiamen University, Zhangzhou, Fujian, China; 3 Department of Gastroenterology, The 909th Hospital, School of Medicine, Xiamen University, Zhangzhou, Fujian, China

**Keywords:** case report, hepatitis B virus, hepatocellular carcinoma, immunotherapy, intrahepatic cholangiocarcinoma, metachronous tumors

## Abstract

We describe a case of metachronous double primary hepatocellular carcinoma (HCC) and intrahepatic cholangiocarcinoma (iCCA) in a 62-year-old male with occult hepatitis B virus (HBV) infection, an extremely rare entity easily misdiagnosed as iCCA recurrence, especially with an alpha-fetoprotein (AFP)-negative phenotype. The patient was initially diagnosed with stage IV iCCA (cT3N0M1) with bone metastases, receiving radiotherapy for primary and metastatic lesions, followed by 2-year sequential apatinib plus camrelizumab, achieving iCCA partial remission. At 56-month follow-up, abdominal CT detected new hepatic lesions, which pathological and immunohistochemical examinations confirmed as *de novo* HCC rather than iCCA recurrence, with the patient remaining AFP-negative throughout the course. We retrospectively analyzed this case and reviewed relevant literature to discuss its diagnostic clues, potential pathogenesis and therapeutic implications. This case highlights that occult HBV infection, chronic inflammation and liver radiotherapy may synergistically drive multi-pathway hepatocarcinogenesis. Radiotherapy combined with anti-angiogenic and immunotherapy is an effective option for advanced unresectable iCCA. It also emphasizes that active pathological biopsy is essential for new hepatic lesions after iCCA remission to clarify diagnosis and avoid misdiagnosis-induced inappropriate treatment.

## Introduction

Hepatocellular carcinoma (HCC) and Intrahepatic cholangiocarcinoma (ICCA) are the two predominant pathological subtypes of primary liver cancer, with substantial variations in cellular origin, molecular carcinogenic mechanisms, clinical characteristics, and therapeutic protocols. ICCA originates from intrahepatic bile duct epithelial cells and is often manifested by elevated carbohydrate antigen 19-9 (CA19-9). Conversely, HCC arises from hepatocytes, closely associated with HBV infection, HCV infection, and cirrhosis, and elevation of alpha-fetoprotein (AFP) is one of its typical clinical indicators (1). A subset of HCC patients present with occult HBV infection. These patients deny a history of hepatitis, but laboratory examinations show negative HBsAg expression and positive HBV-DNA quantification (2).

Synchronous double primary hepatic cancer (DPHC) involving both ICCA and HCC is relatively rare, accounting for only 0.25%–0.26% of all primary liver cancers (3, 4), and metachronous DPHC (sequential occurrence of the two subtypes) is much rarer. Such cases are highly prone to misdiagnosis as tumor recurrence due to a previous ICCA history, leading to inappropriate selection of therapeutic strategies and adverse impacts on prognosis. However, the association between occult HBV infection and the occurrence of DPHC, as well as its impacts on diagnosis and treatment, has not yet been fully elucidated.

Anti-angiogenic targeted therapy and immune checkpoint inhibitors have evolved into a promising therapeutic approach for advanced unresectable intrahepatic cholangiocarcinoma, which can achieve long-term disease control in some patients (5, 6). Yet, the question of whether long-term treatment and related hepatic injury might raise the risk of secondary primary liver cancer (e.g., HCC) remains unresolved. We herein report an uncommon case of a patient with occult HBV infection who developed metachronous HCC after achieving a sustained partial response (PR) of advanced iCCA following radiotherapy combined with apatinib and camrelizumab. Combined with the relevant literature, we discuss the diagnostic pitfalls, potential pathogenesis, and therapeutic implications, aiming to provide a clinical reference for similar practice.

## Case presentations

**Figure 1:**
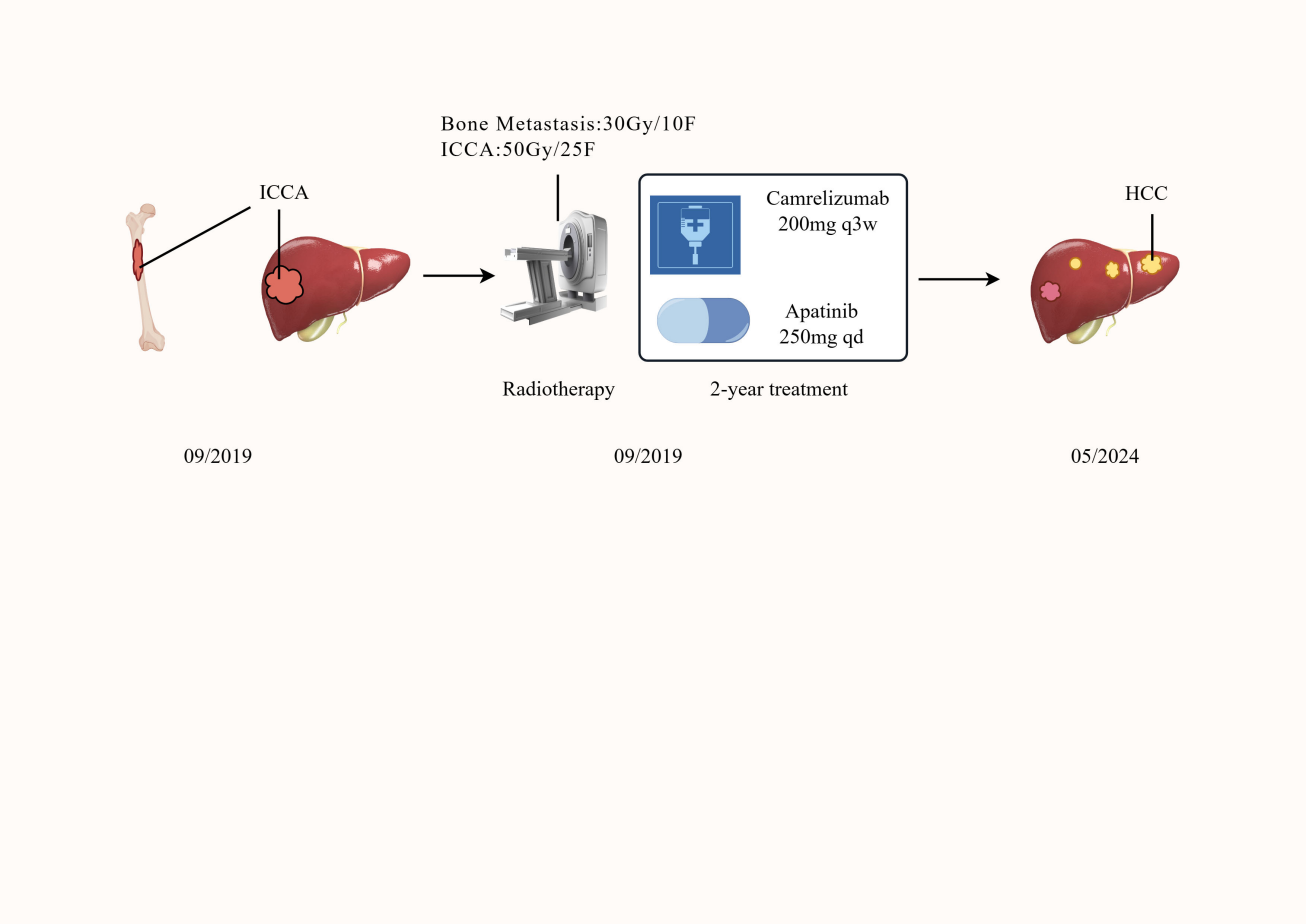
Timeline of the patient’s diagnosis and treatment. Key time points and corresponding events are shown.

The diagnostic and therapeutic timeline of the patients was presented in **Figure 1**. In September 2019, a 62-year-old male presented with right thigh pain of 1 hour in duration, which occurred after a fall. He had a 10−year history of cholelithiasis and denied any history of hepatitis, smoking, alcohol abuse, metabolic syndrome, use of hepatotoxic medications, or family history of cancer. On physical examination, there was tenderness and crepitus at the midshaft of the right femur. X-ray and CT studies showed a pathological fracture of the midshaft of the right femur. During the planned procedure of “open reduction and internal fixation of the right femur”, grayish-white tumor tissue infiltration and osteolytic destruction were identified at the fracture end, consistent with a neoplastic fracture. The operation was therefore changed to “tumor resection and internal fixation of the right femur”. Postoperative pathology demonstrated metastatic adenocarcinoma, and immunohistochemistry was suggestive of a biliary epithelial origin. The patient was referred to the Department of Oncology at our hospital for identification of the primary tumor and comprehensive disease evaluation.

**Figure 2:**
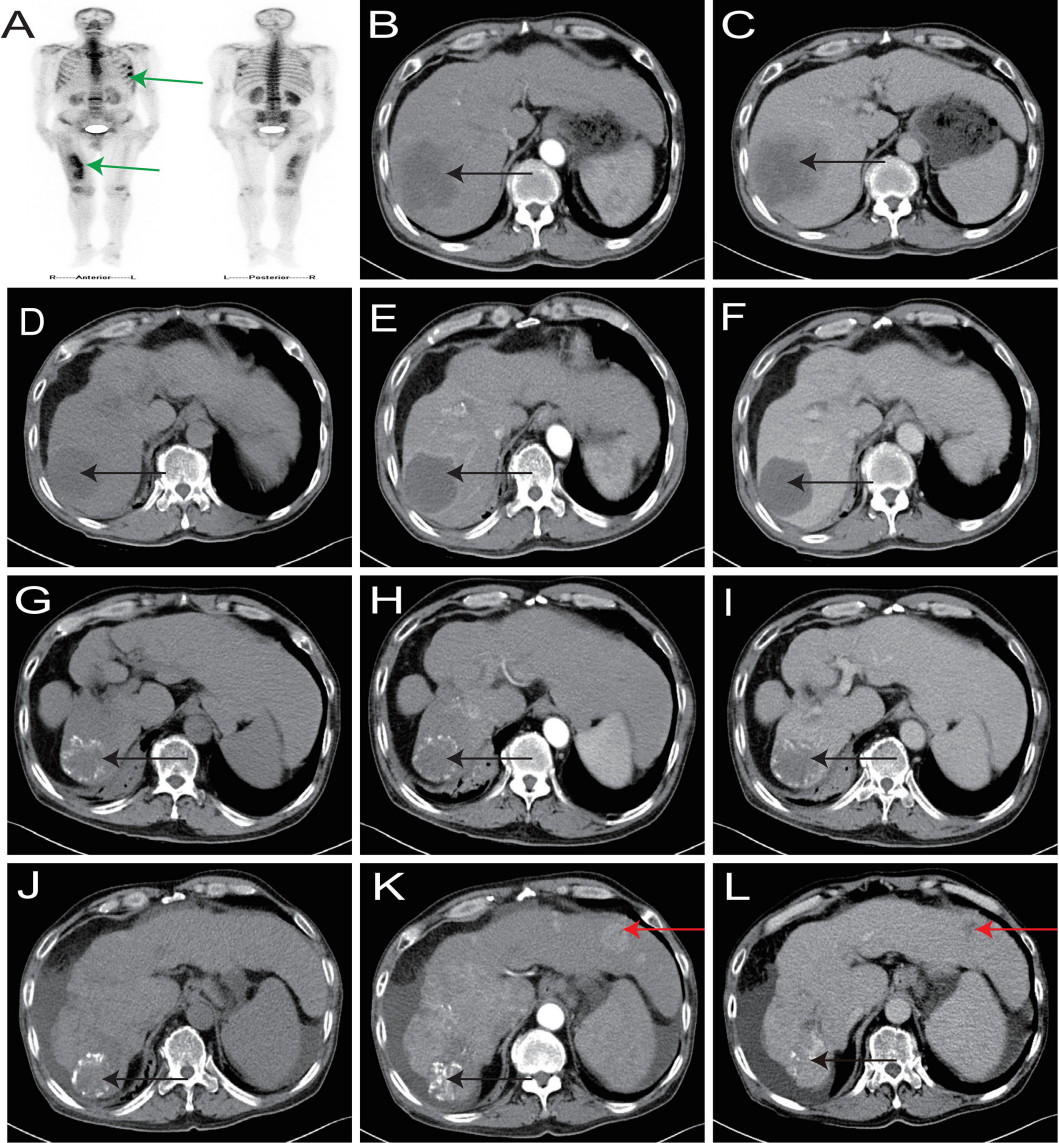
Dynamic changes of target lesions. **(A)** Bone metastatic lesions (green arrows). **(B, C)** Hepatic lesions at initial diagnosis (black arrows). **(D-F)** Shrunken hepatic lesions with liquefactive necrosis at 6 months post-treatment (black arrows) **(G-I).** Shrunken hepatic lesions with necrosis and calcification at 2 years post-treatment (black arrows). **(J-L)** Intrahepatic metastases identified at 56 months after initial diagnosis (red arrows).

**Figure 3:**
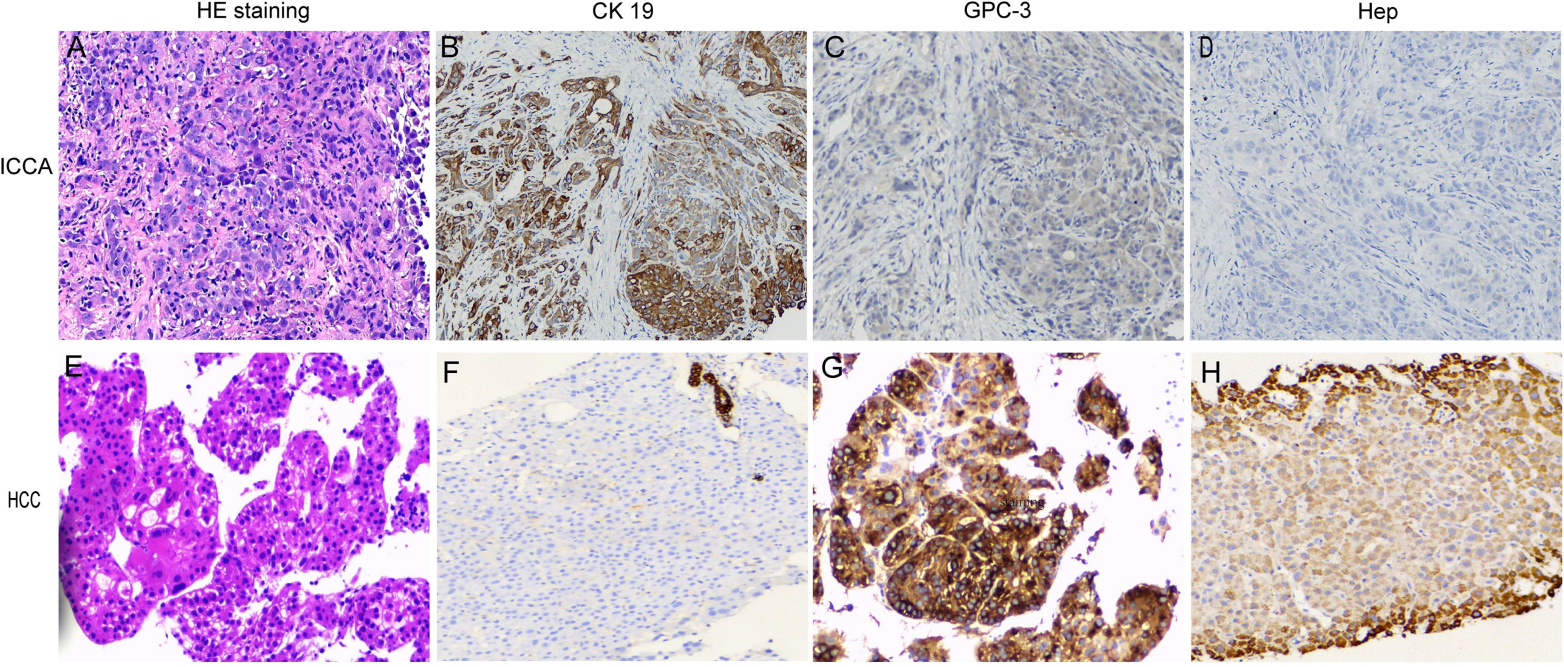
HE staining and immunohistochemical findings (×100). **(A)** HE staining of iCCA: tumor cells arranged in glandular and trabecular patterns with marked nuclear atypia. **(B)** Diffuse positive expression of CK19 in iCCA. **(C, D)** Negative expression of GPC-3 and Hep in iCCA. **(E)** HE staining of HCC: tumor cells arranged in nest and trabecular patterns with invasive growth, round or irregular nuclei and multinucleated giant cells present. **(F)** Negative expression of CK19 in HCC. **(G, H)** Positive expression of GPC-3 and Hep in HCC tumor cells.

Baseline evaluations at admission included a liver CT scan demonstrating a 9.0 cm × 8.3 cm mass in the right hepatic lobe with delayed contrast enhancement (**Figures 2B, C**), with no radiological signs of cirrhosis or splenomegaly; serum albumin 41.3 g/L (reference range: 40–55 g/L) and total bilirubin 9.92 μmol/L (reference range: 0.00–25.00 μmol/L); an AFP level of 2.49 ng/mL (reference range: 0–8.78 ng/mL); a CA19–9 level of 1043.42 U/mL (reference range: 0–37 U/mL); hepatitis B serology revealing HBsAg<0.2 ng/mL (reference range: 0–0.2 ng/mL), HBeAg<0.1 PEIU/mL (reference range: 0–0.7 PEIU/mL), anti-HBe<0.1 PEIU/mL (reference range: 0–2 PEIU/mL), and anti-HBc>60 PEIU/mL (reference range: 0–5.3 PEIU/mL, positive); negative serology for hepatitis A (HAV), hepatitis C (HCV), and hepatitis E (HEV) viruses; and an HBV-DNA load of 1.04×10² IU/mL (high-sensitivity real-time fluorescent quantitative PCR assay, Xi’an Tianlong Technology Co., Ltd.; reference range: <50 IU/mL). Whole-body bone scintigraphy identified metastatic lesions involving the 4th, 5th, and 6th left ribs and the right femur (**Figure 2A**). Histopathological analysis of liver biopsy specimens confirmed iCCA, with tumor cells arranged in trabeculae and exhibiting marked nuclear atypia on hematoxylin-eosin (HE) staining; immunohistochemical (IHC) profiling showed positive cytokeratin 19 (CK19) expression and negative expression of hepatocyte antigen (HEP) and glypican-3 (GPC3) (**Figures 3A–D**). A diagnosis of iCCA cT3N0M1 Stage IV, Child–Pugh class A, and occult HBV infection was made. The patient declined gemcitabine-cisplatin due to fear of toxicities and prioritization of quality of life, opting for a non-chemotherapy regimen. After multidisciplinary review, he consented to radiotherapy plus apatinib-camrelizumab. The primary hepatic lesion was treated with 6 MV X-ray IMRT to a total dose of 50 Gy in 25 fractions to the gross tumor volume (GTV) and 45 Gy in 25 fractions to the planning target volume (PTV) over 5 weeks, and the right femoral metastasis was treated with 30 Gy in 10 fractions over 2 weeks using the same technique. Systemic therapy consisted of apatinib 250 mg once daily and camrelizumab 200 mg every 3 weeks for 2 years, with concurrent entecavir 0.5 mg orally once daily for antiviral prophylaxis. Grade 2 hypertension (CTCAE) occurred during treatment and was well-managed with antihypertensives. Six months after treatment initiation, a partial response (PR) was achieved per the Response Evaluation Criteria in Solid Tumors version 1.1 (RECIST 1.1): the primary hepatic lesion had reduced in size to 4.4 cm × 4.0 cm (**Figures 2D–F**), meeting the PR criteria, with CA19–9 decreased to 19.22 U/mL and AFP remaining within the normal range. Targeted and immunotherapy were discontinued after 2 years of continuous administration because of sustained partial response and financial constraints; re-evaluation confirmed that the hepatic lesions still maintained a PR status per RECIST 1.1 (**Figures 2G-I**), and only antiviral therapy was continued thereafter.

Follow-up continued until May 2024 (56 months following initial presentation), at which time abdominal CT scan identified cirrhosis, splenomegaly, and ascites, as well as multiple round hypodense lesions in the liver (maximum diameter 2.7 cm × 2.3 cm) with a “fast-in and fast-out” enhancement pattern on contrast-enhanced imaging (**Figures 2J–L**). All new lesions were located outside the target volume of previous hepatic radiotherapy.

Laboratory tests showed normal AFP (3.05 ng/mL), markedly elevated PIVKA-II (17654.57 mAU/mL; reference range: 0–40 mAU/mL), normal CA19-9 (32.17 U/mL), decreased serum albumin (29.5 g/L), elevated total bilirubin (62.39 μmol/L), prothrombin time 11.7 seconds (reference range: 9.8–12.1 seconds), and an HBV-DNA load of 1.76×10² IU/mL (reference range: <50 IU/mL). Liver biopsy of the newly developed hepatic lesions confirmed the diagnosis of HCC. On HE staining, the tumor cells exhibited a nested and trabecular pattern with invasive growth, round or irregular nuclei, and identifiable multinucleated giant cells, with negative expression of CK19 and positive expression of HEP and GPC3 (**Figures 3E–H**). A diagnosis of metachronous HCC with intrahepatic metastases was established, with hepatic function graded as Child–Pugh class B.

Following the diagnosis of HCC, clinicians advised continuation of the previous targeted-immunotherapy regimen combined with local ablation for disease control. Unfortunately, due to financial constraints and personal preference, the patient opted to forgo further treatment and was discharged. He succumbed to the disease 2 months after discharge.

## Discussion

The core clinical characteristics and rarity of this case are mainly reflected in several aspects. First, the patient presented with metachronous double primary liver cancer: he was initially diagnosed with Stage IV iCCA accompanied by multiple bone metastases, achieved PR after comprehensive treatment, and subsequently developed metachronous HCC at the 56th month of follow-up after initial diagnosis. The clinical incidence of two distinct pathological types of primary liver cancer occurring sequentially in the same patient is extremely low, with few relevant reports currently available. Second, negative hepatitis B surface antigen (HBsAg) but detectable HBV-DNA, as a potential inducer of double primary liver cancer, further enhances the particularity of this case. Additionally, the patient remained AFP-negative throughout the entire course: from iCCA diagnosis and treatment remission to HCC occurrence, AFP levels were consistently maintained within the normal range, classifying this case as AFP-negative HCC, which accounts for approximately 30% of all HCC cases (7). Compared with AFP-positive HCC, AFP-negative HCC poses greater diagnostic challenges and is prone to delayed diagnosis due to normal AFP levels. Finally, the patient’s treatment course and prognosis were distinctive: after achieving a sustained PR for the iCCA, the primary lesion remained stable without progression or recurrence for the entire follow-up period, yet metachronous HCC developed—an outcome markedly different from the typical clinical course of iCCA recurrence. This unique clinical scenario further alerts clinicians to remain vigilant about the potential development of secondary primary hepatocellular carcinoma during long-term follow-up in patients with iCCA who have achieved a treatment-induced partial response.

The key differential point in this case is distinguishing iCCA recurrence from metachronous HCC. They differ significantly in cellular origin, pathological features, imaging, and tumor marker. From the pathological aspect, immunohistochemistry is the gold standard for the diagnosis of ICCA and HCC: iCCA shows CK19 (+), GPC3 (–), HEP (-), while HCC shows CK19 (-), GPC3 (+), HEP (+). In this case, the two tumors had completely opposite immunophenotypes, clearly confirming metachronous DPHC and ruling out iCCA recurrence (8). Imaging-wise, typical iCCA is hypovascular with delayed enhancement, whereas classic HCC presents with “fast-in and fast-out” enhancement (1). The imaging features of HCC in this case were markedly different from iCCA, further supporting metachronous occurrence. For tumor markers, CA19-9 was normal, whereas PIVKA-II was markedly elevated, supporting HCC. Despite negative AFP, PIVKA-II is a specific HCC biomarker; biopsy remains confirmatory (9). However, a critical uncertainty remains: whether the metachronous lesion represents *de novo* HCC, cHCC-CCA, or a clonally related tumor with lineage plasticity. cHCC-CCA accounts for 0.4 to 14.2% of primary liver malignancies and may present as distinct HCC/CCA components or biphenotypic cells (10). On small biopsies, this entity can mimic pure HCC, and definitive distinction would require NGS, which was not performed due to limited availability and cost; the patient’s subsequent deterioration precluded further retrospective analysis.

The sequential occurrence of iCCA and HCC in this patient might reflect a combination of factors, including occult HBV infection, chronic inflammation secondary to cholelithiasis, and possibly radiotherapy-induced liver injury. HBV infection is the most critical risk factor for HCC; even occult HBV infection markedly elevates HCC risk (11). HBV drives malignant transformation of hepatocytes through genomic integration and HBx protein regulation (12, 13), and persistent intrahepatic inflammation from chronic HBV infection further promotes DNA damage and accelerates malignant transformation (14). Cholelithiasis, a well-recognized risk factor for iCCA, causes chronic bile duct injury that preferentially drives biliary carcinogenesis (15), consistent with the pathogenesis of the initial iCCA in this patient. A contributory role of radiotherapy is speculative. The metachronous HCC developed 54 months after irradiation but outside the high-dose field. Radiation can induce genomic instability (16), and local therapies have been reported to cause lineage changes in experimental settings (17, 18). However, direct evidence linking radiotherapy to this patient’s HCC is lacking; occult HBV infection and chronic inflammation remain the dominant causes. Notably, the transdifferentiation potential between hepatocytes and cholangiocytes has been documented: cholangiocytes can differentiate into functional hepatocytes when hepatocyte regeneration is impaired (19), providing a cellular basis for the pathogenesis of double primary liver cancer. According to the latest NCCN Clinical Practice Guidelines for Biliary Tract Cancers (2026.V1), the preferred first-line regimens for advanced intrahepatic cholangiocarcinoma (ICC) are durvalumab or pembrolizumab combined with gemcitabine and cisplatin (category 1 evidence), with gemcitabine plus cisplatin as the classic chemotherapy backbone. For second-line treatment, FOLFOX is the preferred chemotherapy regimen (category 1 evidence), while targeted therapies (e.g., pemigatinib for FGFR2 fusions, ivosidenib for IDH1 mutations) and immunotherapy are recommended based on molecular profiling (20, 21). Notably, approximately 40%–50% of iCCA cases have targetable molecular alterations (22), whereas targeted therapy for HCC does not require biomarker screening. The addition of an antiangiogenic tyrosine kinase inhibitor (TKI) to ICI-containing chemotherapy may further improve clinical outcomes. In the randomized phase II SAGC trial, sintilimab plus anlotinib plus GC resulted in significantly longer progression-free survival (8.5 vs. 6.3 months; HR 0.48) and OS (17.4 vs. 13.4 months; HR 0.61) compared with GC alone (23).

For patients who refuse or cannot tolerate chemotherapy, ICI-TKI doublet regimens represent a valuable alternative. A phase Ib/II trial of camrelizumab plus apatinib in advanced primary liver cancer, including seven patients with iCCA, demonstrated manageable toxicity and promising antitumor activity (24), another case report and literature review reported that such a combined regimen can achieve significant responses in patients with recurrent iCCA, supporting its clinical application in advanced iCCA (5). In the second-line setting, sintilimab plus anlotinib yielded an objective response rate of 30%, median progression-free survival of 6.5 months, and median OS of 12.3 months (25). The international phase II LEAP-005 study further corroborated the efficacy of lenvatinib plus pembrolizumab in previously treated advanced biliary tract carcinoma, with an objective response rate of 17.6% and favorable safety (26). For treatment-naïve patients with unresectable iCCA, a prospective phase II trial showed that front-line sintilimab plus anlotinib achieved an objective response rate of 33.3%, median progression-free survival of 7.49 months, and a conversion resection rate of 16.7% (27).

The use of apatinib in the present case is further supported by evidence of its single-agent activity in refractory disease. Several retrospective studies conducted between 2017 and 2019 demonstrated that apatinib monotherapy achieved a median PFS of 2.8–4.3 months and median OS of 6.2–8.8 months in patients with advanced biliary tract cancers refractory to first-line chemotherapy (28–30). These findings confirm the meaningful clinical activity and favorable safety profile of apatinib, providing a rational backbone for combination strategies. Notably, the therapeutic advantage of the “radiotherapy + targeted therapy + immunotherapy” triple regimen adopted in this case benefits from the synergistic antitumor effect among the three, a synergistic mechanism that has been confirmed by multiple studies. A phase II trial by Zhu et al. showed that initiating camrelizumab treatment within 7 days after radiotherapy for unresectable iCCA resulted in an objective response rate (ORR) of 61.1% and a median progression-free survival (mPFS) of 12.0 months (31). Liu et al. first reported that SBRT combined with PD-1 inhibitors can induce an abscopal effect in iCCA; the mechanism lies in radiotherapy-induced immunogenic cell death of tumor cells, activation of specific T cell responses, and PD-1 blockade reversing T cell exhaustion to amplify the *in situ* vaccine effect (32).

A recent multi-center real-world study further confirmed that local therapy combined with ICIs and targeted drugs (triple combination therapy) is more effective than immunotherapy combined with chemotherapy in advanced iCCA (33). A study by Xiao et al. confirmed that camrelizumab combined with chemoradiotherapy significantly improved survival in adjuvant treatment of resectable extrahepatic cholangiocarcinoma and gallbladder cancer, further supporting the application value of camrelizumab combined with radiotherapy in biliary tract tumors (34). The results of this study also provide real-world long-term follow-up evidence for the clinical value of this treatment strategy, supporting the feasibility of the “chemotherapy-free” triple regimen in selected iCCA patients. Additionally, in terms of prognosis, the overall prognosis of HCC is better than that of iCCA; however, after progressing to advanced stages (BCLC stage C or metastatic iCCA), there is no significant difference in median overall survival between the two (7.8 months for HCC vs 8.5 months for iCCA), suggesting similar therapeutic dilemmas in the terminal stage. Nevertheless, HCC can be treated sequentially with multiple TKI drugs, resulting in a significantly longer overall treatment management window than iCCA (35).

Several limitations exist in this report. First, a single case cannot exclude accidental correlations, and the causal link between iCCA remission and metachronous HCC requires further verification. Second, next-generation sequencing was not conducted, precluding determination of driver gene status and clonal evolution, which limits molecular exploration of tumor origin. Third, irregular monitoring of HBV markers prevented accurate evaluation of antiviral treatment efficacy. Fourth, follow-up data were limited because the patient declined treatment after HCC diagnosis. Future multi-center studies and multi-omics analyzes are required to elucidate the mechanisms and improve clinical decision-making.

## Conclusion

This case highlights that metachronous iCCA and HCC is diagnostically challenging. HBV infection, chronic inflammation, and liver radiotherapy may contribute to the development of double primary cancers. Pathological confirmation is crucial to avoid misdiagnosing *de novo* HCC as iCCA recurrence.

## Data Availability

The original contributions presented in the study are included in the article/**Supplementary Material**. Further inquiries can be directed to the corresponding authors.
